# Gastroprotective and Antioxidant Effects of Ferula Plant Extract Against Indomethacin Induced Gastric Ulcer in Rats

**DOI:** 10.1002/fsn3.70730

**Published:** 2025-07-30

**Authors:** Serdar Yigit, Fatma Necmiye Kaci, Arzu Gezer, Muhammed Yayla, Lale Duysak, Pınar Aksu Kilicle, Erdem Toktay, Nilnur Eyerci, Gül Esma Akdogan Karadag, Seyit Ali Bingol

**Affiliations:** ^1^ Histology and Embryology Department Faculty of Medicine, Kafkas University Kars Turkey; ^2^ Department of Molecular Biology and Genetics Faculty of Science, Erzurum Technical University Erzurum Turkey; ^3^ Vocational School of Health Services, Ataturk University Erzurum Turkey; ^4^ Pharmacology Department, Faculty of Medicine Selçuk University Konya Turkey; ^5^ Department of Biochemistry Ataturk University Faculty of Pharmacy Erzurum Turkey; ^6^ Department of Biology, Faculty of Sciences and Arts Kafkas University Kars Turkey; ^7^ Department of Medical Biology Faculty of Medicine, Kafkas University Kars Turkey

**Keywords:** *Ferula*, indomethacin, *p53*, *TNF‐α*, ulcer

## Abstract

Indomethacin is a non‐steroidal anti‐inflammatory drug and may cause oxidative damage in the stomach tissue. Scientific studies are carried out to discover alternative bioactive phytocompounds and to reveal herbal products with pharmacological effects. In our study, we investigated whether the Ferula, which is antimicrobial and anti‐inflammatory, used for treatment of erectile dysfunction in men, menopausal disorders, diabetes, and prevention of osteoporosis, is effective in the treatment of gastric ulcer. 36 Sprague–Dawley adolescent male rats were divided into six groups: indomethacin, indomethacin + Famotidine, indomethacin + Ferula 400 mg, indomethacin + Ferula 800 mg, Ferula 800 mg, and healthy. It was determined that ulcerative areas were decreased in the high‐dose group of Ferula extract. SOD, GSH, and CAT levels increased with Ferula extract in 800 mg doses, and MDA levels decreased with Ferula extract in 800 mg doses compared to the indomethacin group. *TNF‐α* and *p53* gene expression levels were decreased in Ferula extract in low doses (600 mg) compared to the indomethacin group. We determined that Ferula was effective in gastric ulcers. *Ferula orientalis* plant extract may be an alternative way to prevent drug‐induced gastric ulcer. This anti‐ulcer effect will be used as a food supplement in the future with further studies.

## Graphical abstract

The graphic abstract suggests that *Ferula orientalis* may protect gastric mucosa with its anti‐inflammatory and antioxidant properties. Therefore, *Ferula orientalis* extract may serve as a potential herbal supplement in the therapy of gastric ulcers.

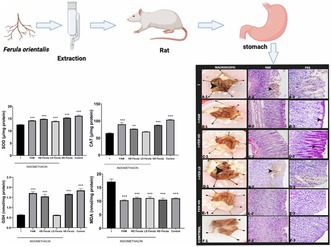

## 1 Introduction

Gastric ulcer occurs as a result of the accumulation of aggressive factors due to insufficient defense mechanisms and these factors degenerating the gastric mucosa. Aggressive factors include hydrochloric acid, pepsin, refluxed bile, leukotrienes, reactive oxygen species (ROS), and so forth (Jagtap and Deore 2018). Ulcer is a phenomenon seen around 10% worldwide (Balaky 2024), especially in the young population. It has a widespread prevalence worldwide and is a serious global health problem affecting approximately the population (Sumbul et al. 2011; Thorsen et al. 2013). Nonsteroidal anti‐inflammatory drugs (NSAID) are the factors that cause gastric ulcer (Keller et al. 2024). NSAIDs are the most prescribed in the community (Vostinaru 2017). When taken at a level that inhibits the formation of prostaglandins (PG), NSAIDs like indomethacin increase mucosal permeability, neutrophil infiltration, oxyradical generation, and ultimately the development of gastric lesions (Takeuchi 2012). In addition, indomethacin, like NSAIDs, has antipyretic, analgesic, and anti‐inflammatory activities (El‐Saghier et al. 2024). To test novel medications' anti‐ulcer qualities, non‐steroidal anti‐inflammatory medications, such as indomethacin, are used to induce ulcers (Satapathy et al. 2024). Its effect mechanism includes the inhibition cyclooxygenase (COX) enzymes and PG synthesis to form gastric ulcer. Indomethacin is used in the treatment of many diseases such as gouty arthritis, traumatic synovitis, osteoarthritis, patent ductus arteriosus, and severe acute respiratory syndrome (Van Overmeire et al. 2000; Amici et al. 2006; Prakash et al. 2009). PGE2 and PGI2 produced by the gastric mucosa play an important role in the protection of mucosal damage (Halter et al. 2001; Konturek et al. 2005). PGs regulate the levels of bicarbonate and mucus secretions with the blood flow and thus protect the stomach from mechanical effects by forming a barrier (Wallace and Devchand 2005; Wallace 2008). Oxidative stress brought on by ulcer‐causing substances damages the mucosa and encourages the development of stomach ulcers (Kumar and Dhiman 2024). Acute inflammation occurs in the ulcer. The ulcer suffers from acute inflammation. One of the most significant pro‐inflammatory cytokines involved in this inflammation is tumor necrosis factor (TNF)‐α (Pradeepkumar Singh et al. 2007) During the ulcer, neutrophil infiltration is observed in the gastric mucosa. Neutrophils react with lipids by producing superoxide radical anion and initiate tissue lipid peroxidation by metabolizing malondialdehyde (MDA) (Kwiecień et al. 2002). Superoxide dismutase (SOD) is one of the enzymes that scavenge reactive oxygen species from the environment and prevent their destructive effects. The function of SOD is to convert O_2_ into the less harmful hydrogen peroxide (H_2_O_2_). The function of catalase (CAT) is to convert H_2_O_2_ into a non‐toxic state. The reduction of H_2_O_2_ to water by glutathione peroxidase is accompanied by the conversion of glutathione from its reduced form (GSH) to its oxidized form (GSSG). When one of these enzymatic events is interrupted, in turn, mucosal damage occurs (Whittle 2003; Lamarque 2004). Ferula orientalis, which is a member of the Apiaceae family, is known as a resin store. Ferula species are called “Caksir”, “Asaotu”, “Kingor” and “Heliz” in Turkish. It has been used as an expectorant, diuretic, carminative, sedative, laxative, and analgesic among the people. It has been reported that Ferula species have coumarins, quinones, flavonoids, and sesquiterpenes (Karakaya et al. 2019). Plant extracts were shown to have a better antifungal impact and good activity against Ferula bacteria, as demonstrated by the antimicrobial analysis, which also revealed that the extracts were generally more effective on the candidates than the test bacteria (Ceylan et al. 2019). Traditional medicine uses Ferula species to cure flatulence and as an anticonvulsant, stimulant, antioxidant, expectorant, and cancer chemotherapy preventative (Razavi et al. 2016). Our study's objective is to show how Ferula orientalis plant extract protects against ulcer damage.

## 2 Material and Method

### 2.1 Plant Extraction

*Ferula orientalis* (asotu, kingor, caksir) used in the study was collected from Şenkaya district of Erzurum in May 2021. In this study, the species identification of the plant was carried out by Dr. Gul Esma Akdogan, who is an expert in the field of botany. However, herbarium registration was not done. It was brought to laboratory and dried there, where there was dry airflow, no direct sunlight, and darkness. The body of the dried samples was ground in a grinder. Ethanol was used as the extraction solvent, and 650 mL of it was put into the boiling flask. Ethanol was preferred as the extraction solvent because it is widely used and has high solvent properties. The Soxhlet method was used because it is the method that is widely used, and the extract yield is obtained most intensively. The solvent was extracted (10–15 siphons) for approximately 10 h until it became clear. The liquid extracts obtained at the end of the 10th hour were filtered through a blue band filter paper so that all particles were removed. The filtered extract sample was evaporated in a rotary evaporator at 35°C–45°C. The plant extract remaining in the balloon was kept in a desiccator for 12 h. The extract, which was completely removed from its solvent, was weighed with an accuracy of 0.1 mg, put into the extract box, and stored at +4°C for use in the study.

### 2.2 Experimental Procedures

We obtained Local Ethics Committee Approval (Permission Number: 2021‐26) before starting the study. For the experiment, 36 Sprague–Dawley adolescent male rats, which were 6 weeks old and 180–200 g, were used. The animals were kept in standard laboratory conditions with 12 h of light and 12 h of darkness at a constant temperature and humidity, standard feeding (ad libitum).

### 2.3 Gastric Ulcer Induction

Animals were fasted for 1 night before indomethacin was used to create the ulcer model. However, water was given during this night. Animals were divided into six groups as indicated below. Low (400 mg/kg) and high (800 mg/kg) doses of Ferula were given orally before 5 min of indomethacin administration. Distilled water was used as a solvent in the control group. Indomethacin at a dose of 25 mg/kg was administered orally by gavage to all groups except groups 5 and 6. All animals were sacrificed taken 6 h after the administration of indomethacin. Thiopental sodium was used to avoid surgical pain. After the stomachs were removed and washed, they were spread over a smooth surface, and the total stomach areas and ulcer areas were measured in square millimeters with the help of millimetric paper.

The following drugs were administered to the rats by gavage to the groups of animals that were fasted 24 h. In dose studies in Ferula species with rats, 400 mg/kg was generally used (Javanshir et al. 2020). We decided to use Ferula extract with 400 mg/kg as a lower dose and 800 mg/kg as a high dose in this study (Table 1).

**TABLE 1:** The descriptions of the experimental groups.

Name	Descriptions	Abbreviations	*n*
Group 1	Distilled water + 25 mg/kg Indomethacin	I	6
Group 2	Indomethacin 25 mg/kg + Famotidine 20 mg/kg	I + FAM	6
Group 3	25 mg/kg Indomethacin + Ferula 400 mg/kg	I + FER LD	6
Group 4	25 mg/kg Indomethacin + Ferula 800 mg/kg	I + FER HD	6
Group 5	Ferula 800 mg/kg	FER HD	6
Group 6	Healthy (Control)	Control	6

Half of the stomach tissues were stored at −80°C and the other half in 10% formalin for histological examination.

### 2.4 Histological Analysis

Taken stomach tissue samples were fixed in 10% formalin solution for 48 h. After fixation, routine tissue follow‐up was performed in accordance with the literature (Sahin et al. 2021). Subsequently, serial sections of 5 μm thickness were taken from each paraffin block for histological examination. Histologically, hematoxylin‐eosin and periodic acid Schiff techniques were performed. Tunnel staining was also performed according to the company's guide (elabscience CAT:E‐CK‐A331). Tissue photographs were taken using the CellSense software program on an Olympus BX43 microscope.

### 2.5 Macroscopic Examination

At the end of our study, the stomachs were cut along the greater curvature, and the gastric lumen was opened in order to determine the ulcerative areas. The ulcerative areas (black colored bleeding foci) were evaluated by using the Image J software. The ulcerated areas in the stomach were seen (Nguelefack et al. 2008; Guha et al. 2010).

### 2.6 Biochemical Analysis

Gastric tissues were ground in liquid nitrogen, weighed to 100 mg, and homogenized in 100 mL phosphate buffer (50 mM, pH 7.4). The homogenates were centrifuged at 4000 rpm for 10 min at 4°C, and the supernatants were collected for experiments.

Gastric tissues were homogenized in phosphate buffer, centrifuged, and the supernatants collected for analysis. Superoxide dismutase (SOD) activity was measured by the reduction of superoxide radicals, with a color change detected at 560 nm, proportional to SOD activity (Sun et al. 1988). Total GSH levels were determined using the Sedlak and Lindsay method, with absorbance measured at 412 nm after incubation (Sedlak and Lindsay 1968). Lipid peroxidation (MDA) was assessed by the formation of a pink complex with thiobarbituric acid at 532 nm following a 60‐min incubation at 95°C (Ohkawa et al. 1979). Catalase activity was measured by incubating supernatant with hydrogen peroxide, stopping the reaction with ammonium molybdate, and measuring absorbance at 405 nm, with activity calculated using a standard curve (Aebi 1984).

### 2.7 RNA Extraction and Reverse‐Transcription Polymerase Chain Reaction Analyses (Rt‐PCR)

Using the total RNA Kit (ECO‐TECH, Erzurum, Turkey) and the manufacturer's recommended procedures, total RNA was extracted from testicular tissue. Nuclease‐free water (NFW) was used to elute the RNA, which was then kept at 80°C. Thermo Scientific's NanoDrop One/Onec Microvolume UV–Vis was used to measure the purity and quantity of total extracted RNA (Thermo Fisher Scientific, Waltham, MA, USA).

The High Capacity cDNA Reverse Transcription kit (Applied Biosystems, Foster City, CA, USA) was used to reverse‐transcribe RNA into cDNA in accordance with the manufacturer's instructions for the reverse‐transcription polymerase chain reaction (Rt‐PCR). Data collection was performed on 7500 Fast Real‐Time PCR (Applied Biosystems). The expression level of each gene was normalized to *β‐Actin* expression level as the reference gene, and relative expression of genes was calculated according to $2^{-∆∆C_{t}}$ method. The difference in gene expressions between groups was calculated by One Way ANOVA using Origin Pro 2024 software (*p* < 0.05).

## 3 Results

### 3.1 Histological Results

**FIGURE 1:**
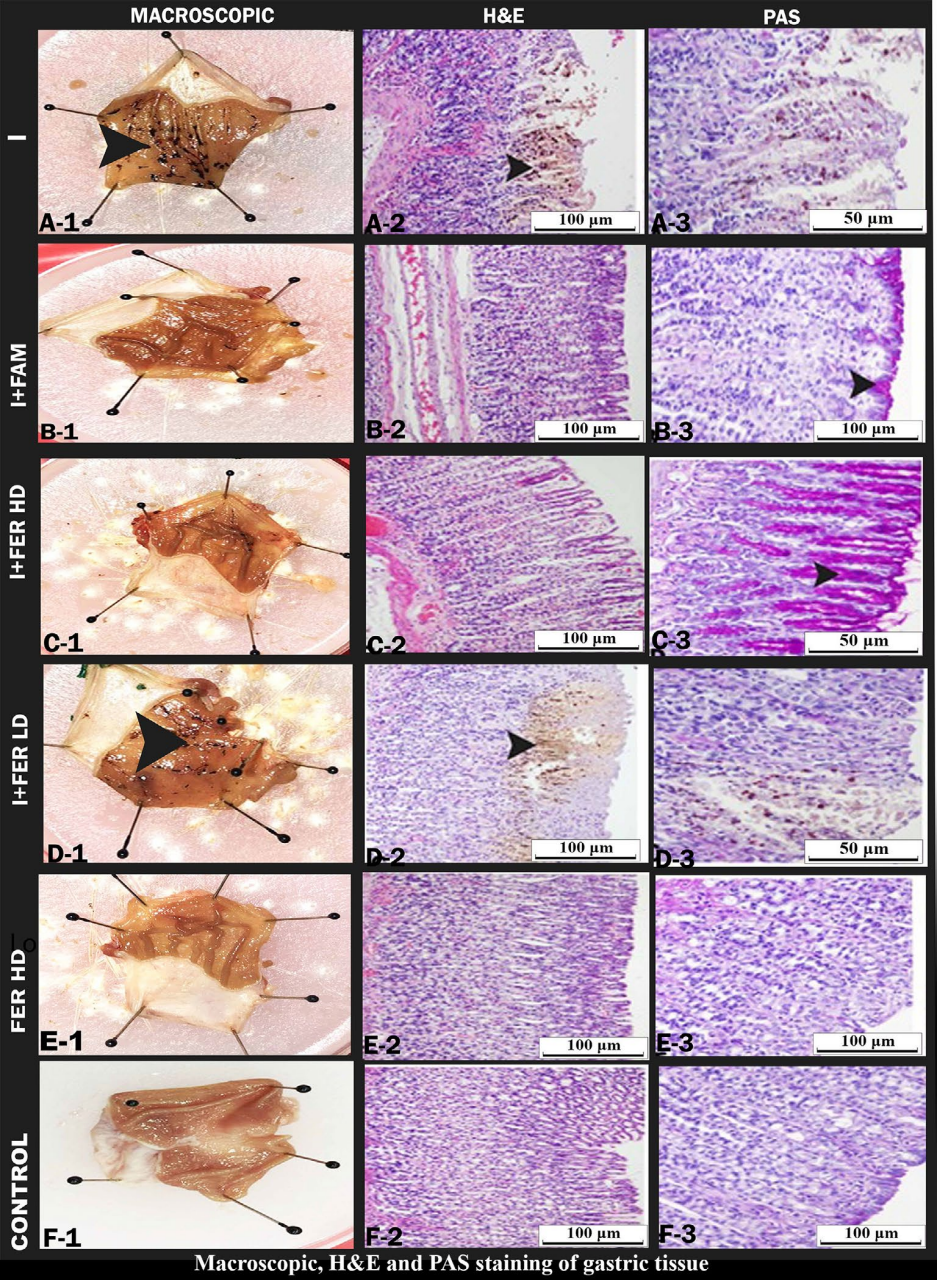
Macroscopic, H&E, and PAS staining of gastric tissue showing ulcer areas and healthy areas. Indomethacin (I) group, A‐1, A‐2, A‐3. Indomethacin + Famotidine (I + FAM) B‐1, B‐2, B‐3. Indomethacin + *Ferula* (400 mg/kg) (I + FERLD) C‐1, C‐2, C‐3. Indomethacin + *Ferula* (800 mg/kg) group (I + FER HD) D‐1, D‐2, D‐3. *Ferula* (800 mg/kg) group (FER HD) E‐1, E‐2, E‐3. Control group (C), F‐1, E‐2, F‐3. Macroscopic A‐1, B‐1, C‐1, D‐1, E‐1, DF‐1, 20× arrowhead macroscopic ulcer areas. Hematoxylin and eosin (H&E) staining A‐2, B‐2, C‐2, D‐2, E‐2, F‐2, 20× arrowhead ulcer areas. PAS staining image A‐3, B‐3, C‐3, D‐3, F‐3, 20× arrow PAS positive cells.

**FIGURE 2:**
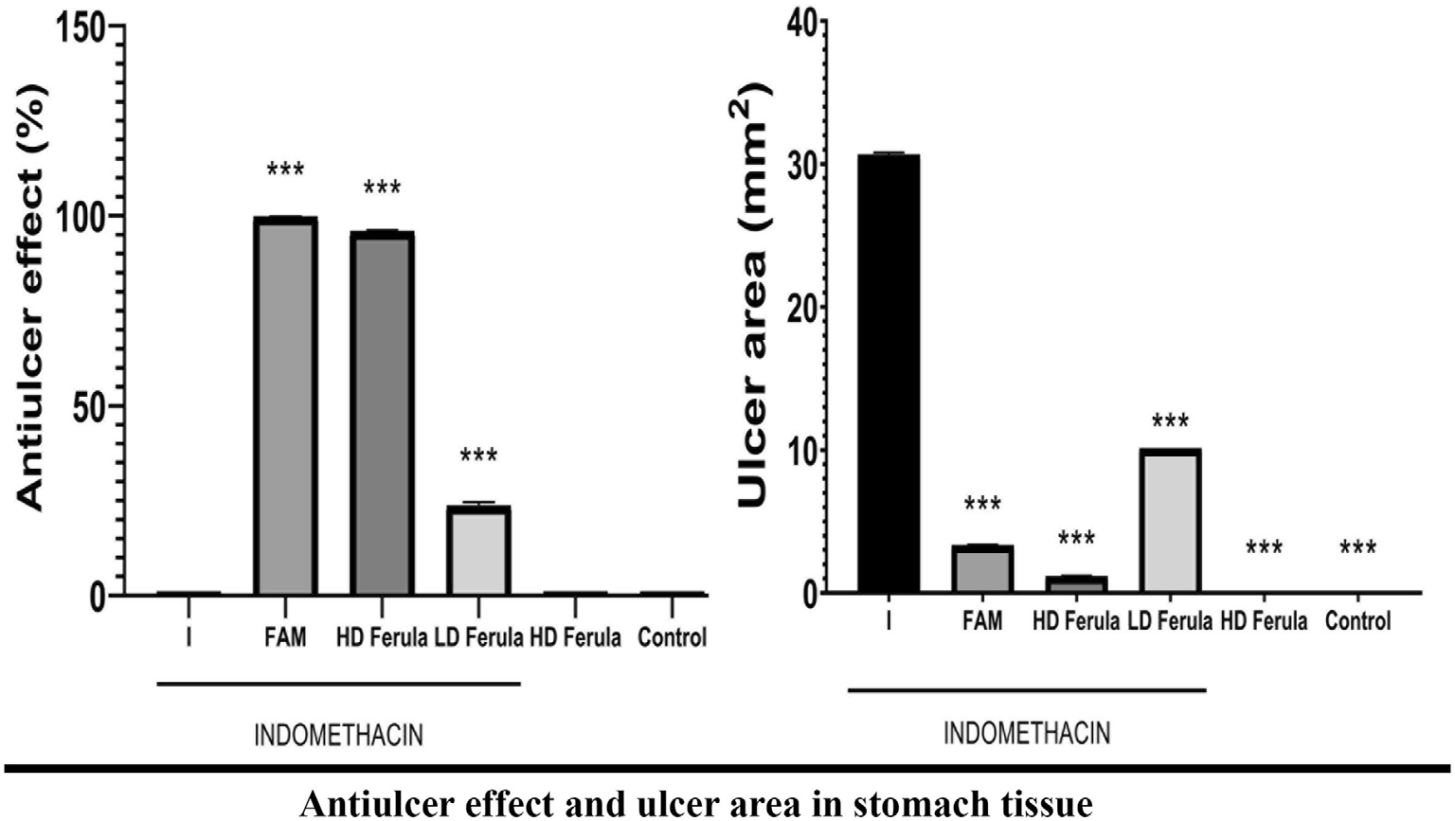
Antiulcer effect and ulcer area in stomach tissue. ***Used to compare with I, used to compare *p* < 0.001.

The graphic illustrates a high correlation between the macroscopic and microscopic data. There were macroscopically identifiable ulcer foci in the indomethacin group's stomachs. Numerous regions of the rats' stomach mucosa in this indomethacin group showed focal or linear black hemorrhages of various diameters. The indomethacin + Ferula (800 mg/kg) group showed signs of bleeding, necrotic regions, and a reduction in the stomach mucus layer (Figure 1). In the INDO+FAM group, no stomach mucosal ulcer focus was seen. It was found that in the group administered plant extract from Ferula orientalis, the anti‐ulcerative effect increased with dose. It was also observed that ulcer areas decreased with increasing dosage. Ferula plant extracts appear to reduce ulcer areas by showing antiulcerative effects on indomethacin‐induced damage in ulcer models (Figure 2). The indomethacin + Ferula orientalis 400 and 800 groups' stomach tissue looked normal. The stomach mucosa of the rats in this group showed a considerable reduction in numerous regions exhibiting visible foci of black ulcers of varied sizes. Edema and necrotic regions were seen to be less prevalent than in the indomethacin group. The number of ulcerated regions in each group of animals' stomach tissues was used to calculate the statistics. In the INDO group, the glycoprotein content decreased due to decreased mucosal production. We observed this as a result of PAS staining. We saw that PAS positive cells increased in the indomethacin + Ferula (800 mg/kg) and Ferula (800 mg/kg) groups.

### 3.2 Biochemical Results

**FIGURE 3:**
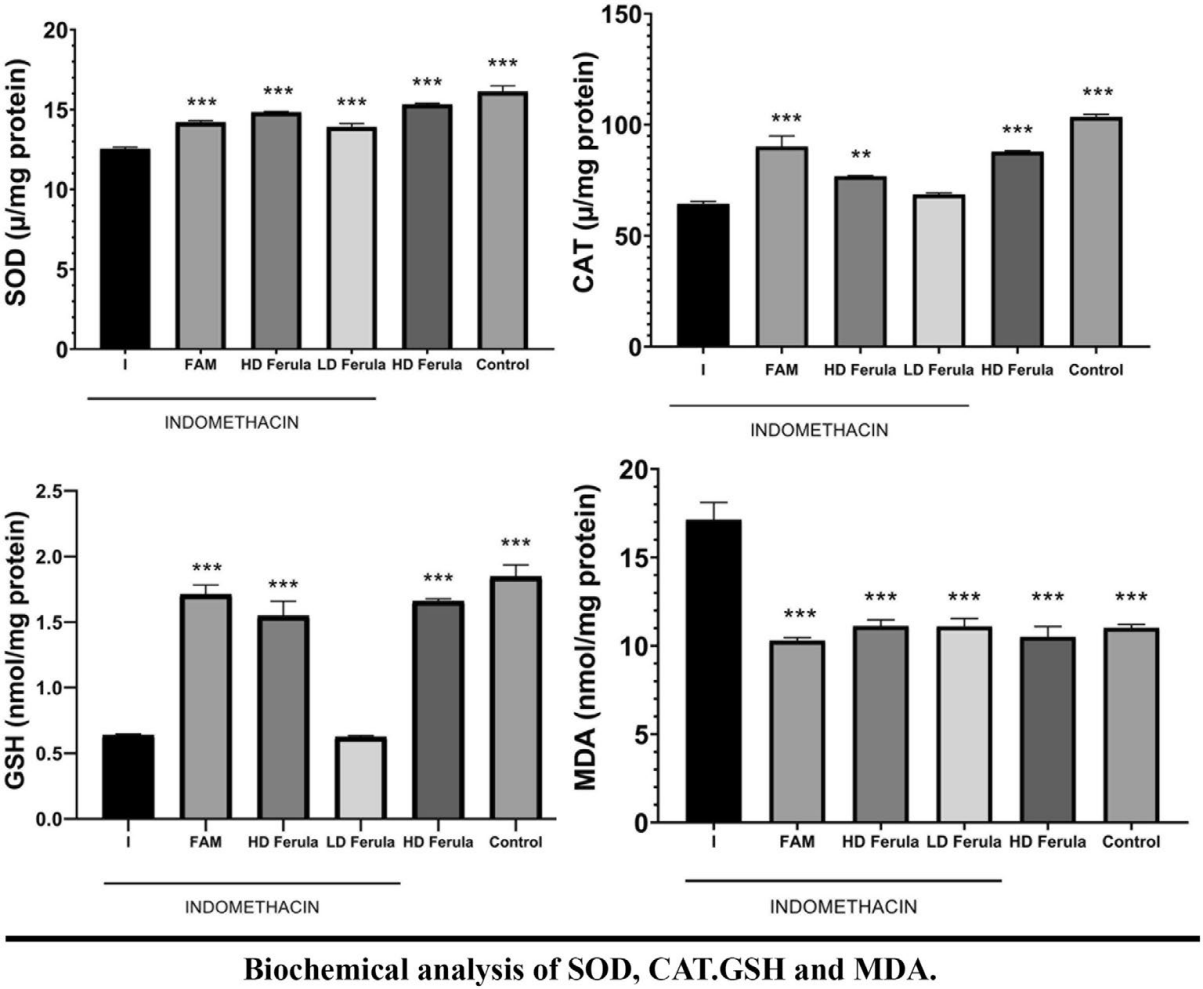
Biochemical analysis of SOD, CAT, GSH, and MDA. ***Used to compare with I, used to compare *p* < 0.05.

In the biochemical analysis, the levels of SOD, CAT, GSH, and MDA in tissues were determined. The lowest level of SOD was found in the indomethacin group. On the other hand, SOD enzyme activity was increased in the groups given indomethacin and Ferula plant extract (Figure 3). According to the statistical results, there was a significant difference between the indomethacin group and Ferula 800 mg/kg and healthy groups in terms of SOD level (*p* < 0.05).

The GSH level of the healthy group was statistically higher than that of the indomethacin group (*p* < 0.05); no significant difference was found between the indomethacin group and other groups (*p* > 0.05) in terms of GSH level. MDA level in the indomethacin group was significantly higher than all other groups (*p* < 0.05). CAT enzyme activity in the indomethacin group was statistically lower than in indomethacin + famotidine, indomethacin + Ferula high‐dose, and healthy groups (*p* < 0.05). CAT enzyme activity in the indomethacin group was found to be similar to the other groups (*p* > 0.05).

### 3.3 RT‐PCR Results

**FIGURE 4:**
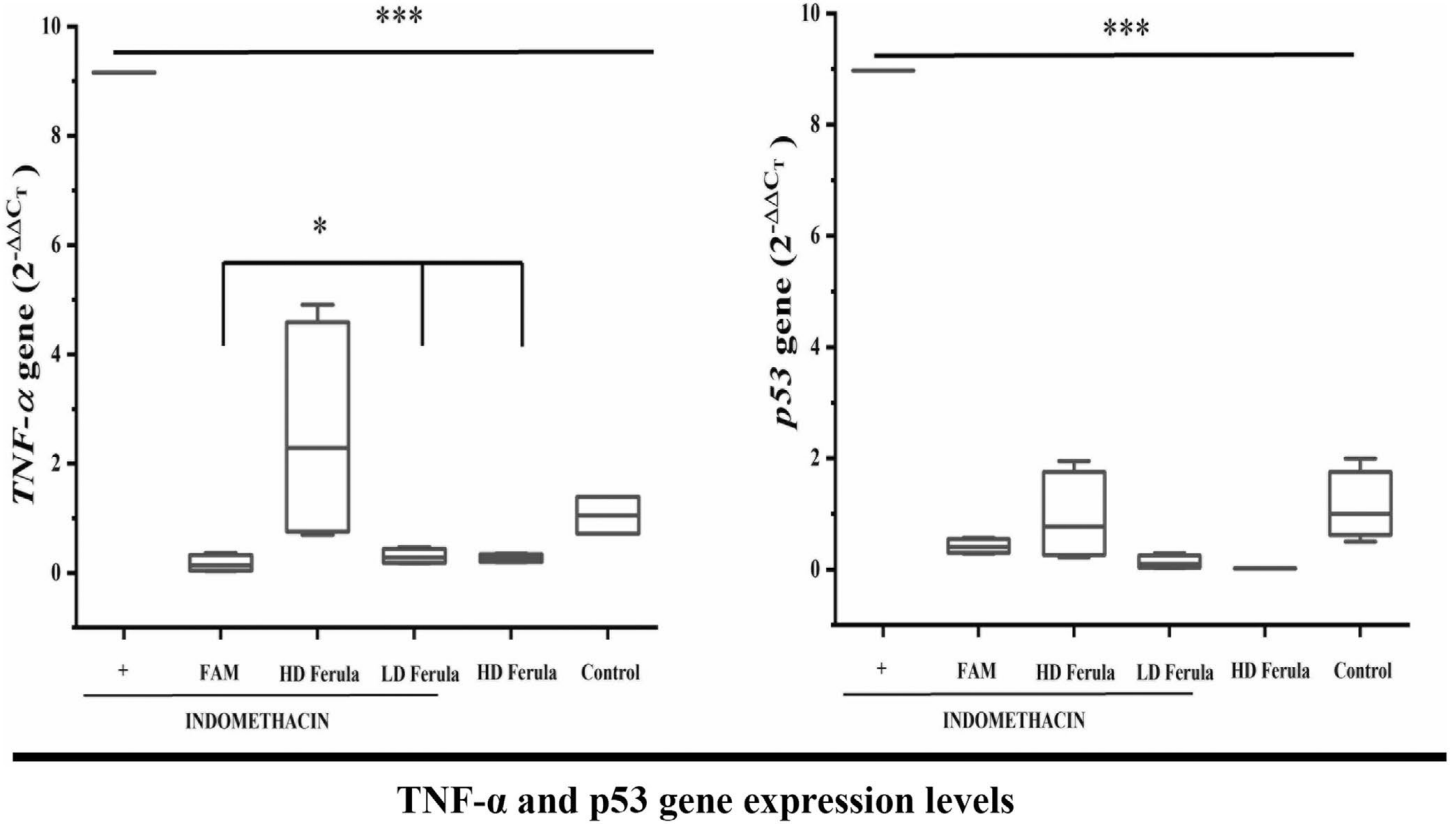
*TNF‐α* and *p53* gene expression levels in indomethacin‐induced gastric ulcer in rats (*p* < 0.001). *Ferula orientalis* administration significantly decreased the expression rates of these two genes (*p <* 0.05).

In our study, mRNA expression levels of *TNF‐α* and *p53* genes were examined. *TNF‐α* and *p53* mRNA expressions in the stomach tissue were found to be significantly higher in the INDO group compared to all other groups (*p* < 0.001). All doses of Ferula orientalis significantly decreased the expression levels of these genes in a dose‐dependent manner (Figure 4). When indomethacin, which is known to increase *TNF‐α* expression, was applied together with *Ferula orientalis*, a decrease was observed in both *TNF‐α* and *p53* gene expression levels. Especially low‐dose application of *Ferula orientalis* statistically significantly decreased the expression levels of both genes (*p* < 0.05).

## 4 Discussion

In this study, we aimed to investigate the protective effect of Ferula plant extract on ulcers induced by indomethacin. Indomethacin, which has a non‐steroidal anti‐inflammatory effect, is a drug used in various inflammatory diseases. This drug is widely used in experimental ulcer studies due to its side effect of ulcers (Singh et al. 2008). In experimental studies, 10–20 and 25 mg/kg doses of indomethacin have been shown to cause ulcers (Süleyman, Demirezer, et al. 2002; Zamora et al. 2008).

In our study, we created an ulcer by applying indomethacin orally at a dose of 25 mg/kg. In ulcer studies, it has been reported that there are serious ulcer areas and lesion areas in the ulcer group (Sathyanarayanan et al. 2022). In the indomethacin‐induced ulcer studies, it was reported that ulcer areas increased compared to other groups (Süleyman, Altinkaynak, et al. 2002). In our study, it is noteworthy that there were many ulcer areas in the ulcer group we created by inducing with indomethacin. It was seen that there were fewer ulcer areas in the indomethacin + Ferula 800 mg/kg group compared to the indomethacin + Ferula 400 mg/kg group. In the previous studies, necrotic tissue loss in the gastric mucosa was noted histopathologically in the group given indomethacin (El‐Ashmawy et al. 2016). In our histologic examination, deformation of epithelial tissue, hemorrhage, degeneration of cells, and cell infiltration were observed in the stomachs of the indomethacin group. Necrotic areas were also observed in the gastric mucosa. This shows that the method in our study is compatible with the method in previous studies.

Studies on Ferula species have shown that they had both antioxidant and gastroprotective effects (Pavlović et al. 2022). In another study, it was mentioned that *Ferula orientalis* had also antioxidant properties (Kızıltaş et al. 2021). In our study, we confirm that the biochemical and histopathological results showed in accordance with the literature in terms of gastroprotective effects.

In the ulcer study, it was reported that while rust positivity decreased in the ulcer group compared to the control group, it increased in the treatment groups (Hassan et al. 2023). As a result of our PAS staining, it was observed that the PAS positive cells increased in the Ferula 800 mg/kg group compared to the indomethacin group. In the cells of aerobic organisms, reactive oxygen species (ROS) are produced as byproducts of regular metabolic processes. The anti‐oxidant defense enzymes, such as malondialdehyde, glutathione peroxidase, superoxide dismutase, and catalase, primarily scavenge these species.

MDA is an important marker of lipid peroxidation in animal studies. It also increases as a result of oxidative damage. In our study, the indomethacin group had the highest MDA level. The Ferula extract, which we gave the animals, decreased the MDA level. We also saw that the extract reduced the oxidative damage depending on the amount of dose. In some ulcer studies, it was found that the MDA level was higher in the indomethacin group and the MDA level decreased by using different extracts (Swarnakar et al. 2005). Some ulcer studies showed that the GSH level decreased in the indomethacin group (Chattopadhyay et al. 2006; Lee et al. 2015). In our study, the GSH level decreased in the indomethacin group like previous studies. It has been reported that SOD enzyme activity decreased in the group given indomethacin (El‐Missiry et al. 2001). In other studies, they found that both SOD and CAT enzyme activities were decreased in the indomethacin group (Alarcón de la Lastra, Nieto, et al. 2002; Djahanguiri 1969; El‐Missiry et al. 2001; Alarcón de la Lastra, Barranco, et al. 2002). However, in some ulcer studies, CAT enzyme activity was found to be high in the indomethacin group (Odabasoglu et al. 2008; Polat et al. 2011). In our study, CAT enzyme activity was the lowest in the indomethacin group, and it was found that the CAT enzyme activity increased in the groups to which we gave indomethacin + Ferula plant extract (*p* < 0.05).

In studies of indomethacin‐induced ulcer, it was reported that the level of *TNF‐α* increased by indomethacin (Cadirci et al. 2010; Wang et al. 2014). It was mentioned that *TNF‐α* is a proapoptotic and cytotoxic cytokine (Sawant et al. 2014). As it is well known, NSAIDs cause stomach damage. This damage is formed through TNF‐α, because increased *TNF‐α* level in gastric tissue damages gastric tissue mucosal cells (Konturek et al. 2000; Baraka et al. 2010) In an ulcer study, it was reported that *TNF‐α* level increased in the indomethacin group when compared to the control group (Thong‐Ngam et al. 2012) In our study, it was observed that *TNF‐α* level expression was highest in the indomethacin group. According to our statistical results, it was determined that there was a significant difference between the other groups and the indomethacin group (*p* < 0.05).

*p53*, an apoptotic marker, carries out cell apoptosis thanks to its broad effect (Yerlikaya et al. 2012). In a study of ulcer induced by indomethacin, it was determined that the *p53* expression level decreased in the indomethacin group (Koriem et al. 2015). However, in another study in which an ulcer was formed with indomethacin, it was found that the *p53* expression level increased in the indomethacin group when it was compared with that of the control group, and *p53* level decreased in the treated group (Abo El Gheit et al. 2020). In our study, it was observed that the *p53* expression level of the indomethacin group was increased, like in the previous study. In addition, the *p53* expression level in Ferula 800 mg/kg + 25 mg/kg indomethacin decreased considerably, and we found a statistically significant difference between this group and the indomethacin group (*p* < 0.05).

## 5 Conclusions

This study investigated the therapeutic effects of Ferula orientalis extract against gastric ulcers. In an indomethacin‐induced gastric injury model, high‐dose administration of Ferula extract significantly reduced ulcerated areas. In addition, Ferula extract at a dose of 800 mg increased the levels of antioxidant enzymes (SOD, GSH, and CAT) and decreased MDA levels compared to the indomethacin group. Low‐dose (400 mg) administration of Ferula significantly down‐regulated the levels of *TNF‐α* and *p53* gene expression. These findings suggest that *Ferula orientalis* may protect the gastric mucosa with its anti‐inflammatory and antioxidant properties. Therefore, *Ferula orientalis* extract may serve as a potential herbal supplement in the therapy of gastric ulcers.

## Author Contributions

**Serdar Yigit: conceptualization (equal), data curation (equal), formal analysis (equal), methodolgy (equal), valitation (equal), writing‐orginal draft (equal). Fatma Necmiye Kaci:** methodology (equal), resources (equal). **Arzu Gezer:** investigation (equal), resources (equal). **Muhammed Yayla:** conceptualization (equal), supervision (equal). **Lale Duysak:** formal analysis (equal), visualization (equal). **Pınar Aksu Kilicle:** formal analysis (equal), resources (equal). **Erdem Toktay:** data curation (equal), resources (equal). **Nilnur Eyerci:** formal analysis (equal), resources (equal). **Gül Esma Akdogan Karadag:** formal analysis (equal), resources (equal). **Seyit Ali Bingol:** formal analysis (equal), resources (equal).

## Ethics Statement

The study was approved by Kafkas University Animal Experiments Local Ethics Committee with decision number 2021‐126.

## Conflicts of Interest

The authors declare no conflicts of interest.

## Data Availability

Data will be made available on request.

## References

[fsn370730-bib-0001] Abo El Gheit, R. E. , M. M. Atef , O. S. El Deeb , et al. 2020. “Unique Novel Role of Adropin in a Gastric Ulcer in a Rotenone‐Induced Rat Model of Parkinson's Disease.” ACS Chemical Neuroscience 11: 3077–3088. 32833426 10.1021/acschemneuro.0c00424

[fsn370730-bib-0002] Aebi, H. 1984. “[13] Catalase in vitro.” In Methods in Enzymology. Elsevier. 10.1016/s0076-6879(84)05016-3 6727660

[fsn370730-bib-0003] Alarcón de la Lastra, C. , M. D. Barranco , M. J. Martín , J. Herrerías , and V. Motilva . 2002. “Extra‐Virgin Olive Oil‐Enriched Diets Reduce Indomethacin‐Induced Gastric Oxidative Damage in Rats.” Digestive Diseases and Sciences 47: 2783–2790. 12498302 10.1023/a:1021025726478

[fsn370730-bib-0004] Alarcón de la Lastra, C. , A. Nieto , M. J. Martín , F. Cabré , J. M. Herrerías , and V. Motilva . 2002. “Gastric Toxicity of Racemic Ketoprofen and Its Enantiomers in Rat: Oxygen Radical Generation and COX‐Expression.” Inflammation Research 51: 51–57. 11930903 10.1007/BF02683999

[fsn370730-bib-0005] Amici, C. , A. Di Caro , A. Ciucci , et al. 2006. “Indomethacin Has a Potent Antiviral Activity Against SARS Coronavirus.” Antiviral Therapy 11: 1021–1030. 17302372

[fsn370730-bib-0006] Balaky, S. T. J. 2024. “Anti *H. pylori*, Anti‐Secretory and Gastroprotective Effects of *Thymus vulgaris* on Ethanol‐Induced Gastric Ulcer in Sprague Dawley Rats.” PLoS One 19: e0287569. 38271407 10.1371/journal.pone.0287569 PMC10810472

[fsn370730-bib-0007] Baraka, A. M. , A. Guemei , and H. A. Gawad . 2010. “Role of Modulation of Vascular Endothelial Growth Factor and Tumor Necrosis Factor‐Alpha in Gastric Ulcer Healing in Diabetic Rats.” Biochemical Pharmacology 79: 1634–1639. 20144589 10.1016/j.bcp.2010.02.001

[fsn370730-bib-0008] Cadirci, E. , B. Z. Altunkaynak , Z. Halici , et al. 2010. “Alpha‐Lipoic Acid as a Potential Target for the Treatment of Lung Injury Caused by Cecal Ligation and Puncture‐Induced Sepsis Model in Rats.” Shock 33: 479–484. 19823117 10.1097/SHK.0b013e3181c3cf0e

[fsn370730-bib-0009] Ceylan, S. , S. Cetin , Y. Camadan , O. Saral , O. Ozsen , and A. Tutus . 2019. “Antibacterial and Antioxidant Activities of Traditional Medicinal Plants From the Erzurum Region of Turkey.” Irish Journal of Medical Science 188: 1303–1309. 30805769 10.1007/s11845-019-01993-x

[fsn370730-bib-0010] Chattopadhyay, I. , U. Bandyopadhyay , K. Biswas , P. Maity , and R. K. Banerjee . 2006. “Indomethacin Inactivates Gastric Peroxidase to Induce Reactive‐Oxygen‐Mediated Gastric Mucosal Injury and Curcumin Protects It by Preventing Peroxidase Inactivation and Scavenging Reactive Oxygen.” Free Radical Biology & Medicine 40: 1397–1408. 16631530 10.1016/j.freeradbiomed.2005.12.016

[fsn370730-bib-0011] Djahanguiri, B. 1969. “The Production of Acute Gastric Ulceration by Indomethacin in the Rat.” Scandinavian Journal of Gastroenterology 4: 265–267. 5346672

[fsn370730-bib-0012] El‐Ashmawy, N. E. , E. G. Khedr , H. A. El‐Bahrawy , and H. M. Selim . 2016. “Nebivolol Prevents Indomethacin‐Induced Gastric Ulcer in Rats.” Journal of Immunotoxicology 13: 580–589. 27224860 10.3109/1547691X.2016.1142488

[fsn370730-bib-0013] El‐Missiry, M. A. , I. H. El‐Sayed , and A. I. Othman . 2001. “Protection by Metal Complexes With SOD‐Mimetic Activity Against Oxidative Gastric Injury Induced by Indomethacin and Ethanol in Rats.” Annals of Clinical Biochemistry 38: 694–700. 11732653 10.1258/0004563011900911

[fsn370730-bib-0014] El‐Saghier, A. M. , S. S. Enaili , A. Abdou , A. M. Hamed , and A. M. Kadry . 2024. “An Operationally Simple, One‐Pot, Convenient Synthesis, and In Vitro Anti‐Inflammatory Activity of Some New Spirotriazolotriazine Derivatives.” Journal of Heterocyclic Chemistry 61: 146–162.

[fsn370730-bib-0015] Guha, P. , A. Dey , A. Chatterjee , S. Chattopadhyay , and S. K. Bandyopadhyay . 2010. “Pro‐Ulcer Effects of Resveratrol in Mice With Indomethacin‐Induced Gastric Ulcers Are Reversed by L‐Arginine.” British Journal of Pharmacology 159: 726–734. 20067468 10.1111/j.1476-5381.2009.00572.x PMC2828036

[fsn370730-bib-0016] Halter, F. , A. S. Tarnawski , A. Schmassmann , and B. M. Peskar . 2001. “Cyclooxygenase 2‐Implications on Maintenance of Gastric Mucosal Integrity and Ulcer Healing: Controversial Issues and Perspectives.” Gut 49: 443–453. 11511570 10.1136/gut.49.3.443 PMC1728453

[fsn370730-bib-0017] Hassan, H. M. , N. M. Alatawi , A. Bagalagel , et al. 2023. “Genistein Ameliorated Experimentally Induced Gastric Ulcer in Rats via Inhibiting Gastric Tissues Fibrosis by Modulating Wnt/β‐Catenin/TGF‐β/PKB Pathway.” Redox Report 28: 2218679. 37260037 10.1080/13510002.2023.2218679 PMC10236962

[fsn370730-bib-0018] Jagtap, M. J. , and A. B. Deore . 2018. “Antiulcer Activity of Methanolic Extract of Roots of *Beta vulgaris*, Chenopodiaceae.” International Journal of Pharmaceutical Sciences and Drug Research 10: 454–459.

[fsn370730-bib-0019] Javanshir, S. , M. Soukhtanloo , M. Jalili‐Nik , A. J. Yazdi , M. S. Amiri , and A. Ghorbani . 2020. “Evaluation Potential Antidiabetic Effects of *Ferula latisecta* in Streptozotocin‐Induced Diabetic Rats.” Journal of Pharmacopuncture 23: 158–164. 33072413 10.3831/KPI.2020.23.3.158 PMC7540228

[fsn370730-bib-0020] Karakaya, S. , G. Göger , F. D. Bostanlik , B. Demirci , H. Duman , and C. S. Kiliç . 2019. “Comparison of the Essential Oils of *Ferula orientalis* L., *Ferulago sandrasica* Peşmen and Quézel, and *Hippomarathrum microcarpum* Petrov and Their Antimicrobial Activity.” Turkish Journal of Pharmaceutical Sciences 16: 69–75. 32454698 10.4274/tjps.77200 PMC7227976

[fsn370730-bib-0021] Keller, C. L. , N. T. Jones , R. B. Abadie , et al. 2024. “Non‐Steroidal Anti‐Inflammatory Drug (NSAID)‐, Potassium Supplement‐, Bisphosphonate‐, and Doxycycline‐Mediated Peptic Ulcer Effects: A Narrative Review.” Cureus 16: e51894. 38333496 10.7759/cureus.51894 PMC10849936

[fsn370730-bib-0022] Kızıltaş, H. , A. H. M. E. T. C. E. Y. H. A. N. Gören , Z. Bingol , S. H. Alwasel , and İ. Gülçin . 2021. “Anticholinergic, Antidiabetic and Antioxidant Activities of *Ferula orientalis* L. Determination of Its Polyphenol Contents by LC‐HRMS.” Records of Natural Products 15: 513–528.

[fsn370730-bib-0023] Konturek, P. C. , A. Duda , T. Brzozowski , et al. 2000. “Activation of Genes for Superoxide Dismutase, Interleukin‐1beta, Tumor Necrosis Factor‐Alpha, and Intercellular Adhesion Molecule‐1 During Healing of Ischemia‐Reperfusion‐Induced Gastric Injury.” Scandinavian Journal of Gastroenterology 35: 452–463. 10868446 10.1080/003655200750023697

[fsn370730-bib-0024] Konturek, S. J. , P. C. Konturek , and T. Brzozowski . 2005. “Prostaglandins and Ulcer Healing.” Journal of Physiology and Pharmacology 56, no. Suppl 5: 5–31. 16247187

[fsn370730-bib-0025] Koriem, K. M. , I. B. Gad , and Z. K. Nasiry . 2015. “Protective Effect of *Cupressus sempervirens* Extract Against Indomethacin‐Induced Gastric Ulcer in Rats.” Interdisciplinary Toxicology 8: 25–34. 27486357 10.1515/intox-2015-0006 PMC4961923

[fsn370730-bib-0026] Kumar, S. , and M. Dhiman . 2024. “ *Helicobacter pylori* Secretary Proteins‐Induced Oxidative Stress and Its Role in NLRP3 Inflammasome Activation.” Cellular Immunology 399: 104811. 38518686 10.1016/j.cellimm.2024.104811

[fsn370730-bib-0027] Kwiecień, S. , T. Brzozowski , and S. J. Konturek . 2002. “Effects of Reactive Oxygen Species Action on Gastric Mucosa in Various Models of Mucosal Injury.” Journal of Physiology and Pharmacology 53: 39–50. 11939718

[fsn370730-bib-0028] Lamarque, D. 2004. “Pathogenesis of Gastroduodenal Lesions Induced by Non‐Steroidal Anti‐Inflammatory Drugs.” Gastroentérologie Clinique et Biologique 28 Spec No 3: C18–C26. 15366671 10.1016/s0399-8320(04)95275-x

[fsn370730-bib-0029] Lee, I. C. , H. S. Baek , S. H. Kim , et al. 2015. “Effect of Diallyl Disulfide on Acute Gastric Mucosal Damage Induced by Alcohol in Rats.” Human & Experimental Toxicology 34: 227–239. 24972622 10.1177/0960327114537095

[fsn370730-bib-0030] Nguelefack, T. B. , C. B. Feumebo , G. Ateufack , et al. 2008. “Anti‐Ulcerogenic Properties of the Aqueous and Methanol Extracts From the Leaves of *Solanum torvum* Swartz (Solanaceae) in Rats.” Journal of Ethnopharmacology 119: 135–140. 18602980 10.1016/j.jep.2008.06.008

[fsn370730-bib-0031] Odabasoglu, F. , Z. Halici , A. Cakir , et al. 2008. “Beneficial Effects of Vegetable Oils (Corn, Olive and Sunflower Oils) and Alpha‐Tocopherol on Anti‐Inflammatory and Gastrointestinal Profiles of Indomethacin in Rats.” European Journal of Pharmacology 591: 300–306. 18621042 10.1016/j.ejphar.2008.06.075

[fsn370730-bib-0032] Ohkawa, H. , N. Ohishi , and K. Yagi . 1979. “Assay for Lipid Peroxides in Animal Tissues by Thiobarbituric Acid Reaction.” Analytical Biochemistry 95: 351–358. 36810 10.1016/0003-2697(79)90738-3

[fsn370730-bib-0033] Pavlović, I. , M. Radenković , S. Branković , et al. 2022. “Spasmolytic, Gastroprotective and Antioxidant Activities of Dry Methanol Extract of *Ferula heuffelii* Underground Parts.” Chemistry & Biodiversity 19: e202200047. 35316577 10.1002/cbdv.202200047

[fsn370730-bib-0034] Polat, B. , Y. Albayrak , B. Suleyman , et al. 2011. “Antiulcerative Effect of Dexmedetomidine on Indomethacin‐Induced Gastric Ulcer in Rats.” Pharmacological Reports 63: 518–526. 21602607 10.1016/s1734-1140(11)70518-7

[fsn370730-bib-0035] Pradeepkumar Singh, L. , P. Kundu , K. Ganguly , A. Mishra , and S. Swarnakar . 2007. “Novel Role of Famotidine in Downregulation of Matrix Metalloproteinase‐9 During Protection of Ethanol‐Induced Acute Gastric Ulcer.” Free Radical Biology & Medicine 43: 289–299. 17603938 10.1016/j.freeradbiomed.2007.04.027

[fsn370730-bib-0036] Prakash, S. , M. Husain , D. S. Sureka , N. P. Shah , and N. D. Shah . 2009. “Is There Need to Search for Alternatives to Indomethacin for Hemicrania Continua? Case Reports and a Review.” Journal of the Neurological Sciences 277: 187–190. 19041987 10.1016/j.jns.2008.10.027

[fsn370730-bib-0037] Razavi, S. M. , L. Nahar , H. Talischi , and S. D. Sarker . 2016. “Ferulone A and Ferulone B: Two New Coumarin Esters From *Ferula orientalis* L. Roots.” Natural Product Research 30: 2183–2189. 26988734 10.1080/14786419.2016.1155574

[fsn370730-bib-0039] Sahin, L. , E. Toktay , M. Yayla , et al. 2021. “The Effect of Dragon Fruit Extract on Experimental Mesentery Arterial Ischemia‐Reperfusion in Rats.” Kafkas Üniversitesi Veteriner Fakültesi Dergisi 27, no. 5: 575–581.

[fsn370730-bib-0040] Satapathy, T. , K. Sen , S. Sahu , et al. 2024. “Experimental Animal Models for Gastric Ulcer/Peptic Ulcer: An Overview.” Journal of Drug Delivery and Therapeutics 14: 182–192.

[fsn370730-bib-0041] Sathyanarayanan, S. , P. S. Sreeja , K. Arunachalam , and T. Parimelazhagan . 2022. “Toxicity and Antiulcer Properties of Ipomoea Wightii (Wall.) Choisy Leaves: An In Vivo Approach Using Wistar Albino Rats.” Evidence‐Based Complementary and Alternative Medicine 2022: 4328571. 35646149 10.1155/2022/4328571 PMC9132668

[fsn370730-bib-0042] Sawant, D. A. , R. L. Wilson , B. Tharakan , H. W. Stagg , F. A. Hunter , and E. W. Childs . 2014. “Tumor Necrosis Factor‐α‐Induced Microvascular Endothelial Cell Hyperpermeability: Role of Intrinsic Apoptotic Signaling.” Journal of Physiology and Biochemistry 70: 971–980. 25392259 10.1007/s13105-014-0366-8

[fsn370730-bib-0043] Sedlak, J. , and R. H. Lindsay . 1968. “Estimation of Total, Protein‐Bound, and Nonprotein Sulfhydryl Groups in Tissue With Ellman's Reagent.” Analytical Biochemistry 25: 192–205. 4973948 10.1016/0003-2697(68)90092-4

[fsn370730-bib-0044] Singh, S. , A. Khajuria , S. C. Taneja , et al. 2008. “The Gastric Ulcer Protective Effect of Boswellic Acids, a Leukotriene Inhibitor From *Boswellia serrata* , in Rats.” Phytomedicine 15: 408–415. 18424019 10.1016/j.phymed.2008.02.017

[fsn370730-bib-0045] Süleyman, H. , K. Altinkaynak , F. Göçer , et al. 2002. “Effect of Nimesulide on the Indomethacin‐ and Ibuprofen‐Induced Ulcer in Rat Gastric Tissue.” Polish Journal of Pharmacology 54: 255–259. 12398157

[fsn370730-bib-0046] Süleyman, H. , L. O. Demirezer , A. Kuruüzüm‐Uz , and F. Akçay . 2002. “Gastroprotective and Antiulcerogenic Effects of *Rumex patientia* L. Extract.” Pharmazie 57: 204–205. 11933853

[fsn370730-bib-0047] Sumbul, S. , M. A. Ahmad , A. Mohd , and A. Mohd . 2011. “Role of Phenolic Compounds in Peptic Ulcer: An Overview.” Journal of Pharmacy & Bioallied Sciences 3: 361–367. 21966156 10.4103/0975-7406.84437 PMC3178942

[fsn370730-bib-0048] Sun, Y. , L. W. Oberley , and Y. Li . 1988. “A Simple Method for Clinical Assay of Superoxide Dismutase.” Clinical Chemistry 34: 497–500. 3349599

[fsn370730-bib-0049] Swarnakar, S. , K. Ganguly , P. Kundu , A. Banerjee , P. Maity , and A. V. Sharma . 2005. “Curcumin Regulates Expression and Activity of Matrix Metalloproteinases 9 and 2 During Prevention and Healing of Indomethacin‐Induced Gastric Ulcer.” Journal of Biological Chemistry 280: 9409–9415. 15615723 10.1074/jbc.M413398200

[fsn370730-bib-0050] Takeuchi, K. 2012. “Pathogenesis of NSAID‐Induced Gastric Damage: Importance of Cyclooxygenase Inhibition and Gastric Hypermotility.” World Journal of Gastroenterology: WJG 18: 2147. 22611307 10.3748/wjg.v18.i18.2147 PMC3351764

[fsn370730-bib-0051] Thong‐Ngam, D. , S. Choochuai , S. Patumraj , M. Chayanupatkul , and N. Klaikeaw . 2012. “Curcumin Prevents Indomethacin‐Induced Gastropathy in Rats.” World Journal of Gastroenterology 18: 1479–1484. 22509079 10.3748/wjg.v18.i13.1479 PMC3319943

[fsn370730-bib-0052] Thorsen, K. , J. A. Søreide , J. T. Kvaløy , T. Glomsaker , and K. Søreide . 2013. “Epidemiology of Perforated Peptic Ulcer: Age‐ and Gender‐Adjusted Analysis of Incidence and Mortality.” World Journal of Gastroenterology 19: 347–354. 23372356 10.3748/wjg.v19.i3.347 PMC3554818

[fsn370730-bib-0053] Van Overmeire, B. , K. Smets , D. Lecoutere , et al. 2000. “A Comparison of Ibuprofen and Indomethacin for Closure of Patent Ductus Arteriosus.” New England Journal of Medicine 343: 674–681. 10974130 10.1056/NEJM200009073431001

[fsn370730-bib-0054] Vostinaru, O. 2017. Adverse Effects and Drug Interactions of the Non‐Steroidal Anti‐Inflammatory Drugs, 17–31. InTech Open.

[fsn370730-bib-0055] Wallace, J. L. 2008. “Prostaglandins, NSAIDs, and Gastric Mucosal Protection: Why Doesn't the Stomach Digest Itself?” Physiological Reviews 88: 1547–1565. 18923189 10.1152/physrev.00004.2008

[fsn370730-bib-0056] Wallace, J. L. , and P. R. Devchand . 2005. “Emerging Roles for Cyclooxygenase‐2 in Gastrointestinal Mucosal Defense.” British Journal of Pharmacology 145: 275–282. 15778736 10.1038/sj.bjp.0706201 PMC1576151

[fsn370730-bib-0057] Wang, T. , S. Zhao , Y. Wang , et al. 2014. “Protective Effects of Escin Against Indomethacin‐Induced Gastric Ulcer in Mice.” Toxicology Mechanisms and Methods 24: 560–566. 25137224 10.3109/15376516.2014.951815

[fsn370730-bib-0058] Whittle, B. J. 2003. “Gastrointestinal Effects of Nonsteroidal Anti‐Inflammatory Drugs.” Fundamental & Clinical Pharmacology 17: 301–313. 12803569 10.1046/j.1472-8206.2003.00135.x

[fsn370730-bib-0059] Yerlikaya, A. , E. Okur , and E. Ulukaya . 2012. “The p53‐Independent Induction of Apoptosis in Breast Cancer Cells in Response to Proteasome Inhibitor Bortezomib.” Tumour Biology 33: 1385–1392. 22477712 10.1007/s13277-012-0386-3

[fsn370730-bib-0060] Zamora, Z. , R. González , D. Guanche , et al. 2008. “Ozonized Sunflower Oil Reduces Oxidative Damage Induced by Indomethacin in Rat Gastric Mucosa.” Inflammation Research 57: 39–43. 18209964 10.1007/s00011-007-7034-1

